# Co-cultured sensory neuron classification using extracellular electrophysiology and machine learning approaches for enhancing analgesic screening

**DOI:** 10.1088/1741-2552/ae0eef

**Published:** 2025-10-23

**Authors:** Alexander Somers, Bryan James Black

**Affiliations:** Department of Biomedical Engineering, Francis College of Engineering, University of Massachusetts Lowell, 1 University Ave, Lowell, MA 01854, United States of America

**Keywords:** hiPSC sensory neurons, nociceptor pharmacology, machine learning

## Abstract

*Objective.* Chronic pain affects over 20% of the adult population in the United States, posing a substantial personal as well as economic burden and contributing to the ongoing opioid crisis. Effective, non-addictive chronic pain treatments are urgently needed. Traditional drug discovery methods have failed to identify novel, non-addictive compounds, highlighting the need for alternative approaches such as phenotypic screening. Our lab developed a phenotypic screening assay using extracellular electrophysiological recordings from co-cultures of human induced pluripotent stem cell sensory neurons and glia. This study aimed to identify responsive neuronal subtypes within these presumptively heterogeneous cultures. *Approach.* We induced an inflammation-like state using tumor necrosis factor alpha and evaluated acute responses to nociceptor agonist capsaicin, which targets transient receptor potential vanilloid-1. By employing unsupervised learning, we labeled responsive cells based on changes in mean firing rates (MFR). We then used the labeled cells’ baseline activity to train and validate five classifiers*. Main results.* None of the classifiers outperformed the others in regards to accuracy. Nonetheless, an RUS-boosted ensemble of decision trees achieved an AUC-ROC of 0.877 classifying nociceptors in an unseen labeled culture. *Significance*. The notable accuracy suggests that machine learning techniques could be employed to enhance microelectrode array-based neuronal phenotypic assays as readouts (e.g. MFR) can be weighted based on target cell type (e.g. nociceptors).

## 1. Introduction

Chronic pain is one of the largest health crises in the United States, affecting over 20% of the adult population [1] and costing the U.S. economy more than $500 billion per year in medical costs and lost productivity [2]. Additionally, the opioid crisis—now primarily fueled by street synthetics, is still affecting millions of Americans each year [3]. More effective and non-addictive analgesics are desperately needed, since classic ‘druggable’ target and drug discovery workflows have been largely ineffective at identifying novel compounds. This is presumably due to the mechanistic complexity of chronic pain pathology. Phenotypic screening is an alternative drug discovery pipeline that does not require complete understanding of the pathophysiological pathways and may be especially useful with complex conditions such as chronic pain.

Recently, our lab demonstrated a novel phenotypic screening assay for inflammatory nociception based on extracellular electrophysiological recordings from co-cultures of human induced pluripotent stem cell (hiPSC) sensory neuron (SN) and glia [4]. This assay resulted in *Z*’ scores suggesting an excellent stand-alone assay based on changes in well-wide extracellular action potential (EAP) spike counts alone. However, spike counts (a widely adopted metric in microelectrode array (MEA) studies) is not only an averaged, but an aggregated metric. The recording radius of a single recording electrode is approximately 20–100 *µ*m [5], meaning that several neurons may be recorded by a single electrode, all contributing to the electrode- and well-wide spike count. More importantly, SN cultures—primary rodent, primary human, and hiPSC—are highly heterogeneous [6, 7], exhibiting differential expression of transmembrane receptors [8], G-coupled protein receptors [9], voltage- and ligand-gated ion channels [10], and secreted markers [11]. Each of the aforementioned biomarkers influences spontaneous and/or evoked activity as well as pharmacological response. Therefore, a given compound may be highly effective with one or more SN subtypes, but the effect may be averaged out at the well-wide level. For novel analgesic screening, it may be advantageous to target SN types that are primarily responsible for detecting noxious stimuli, called ‘nociceptors’. At this point, there are at least two options. (1) Purify the cultures for a down- selected subtype before seeding or (2) retain the inherent heterogeneity in the culture model and develop ways to analyze subtypes that is or is not responsive to a given treatment. This work adopts the second option.

**Figure 1.**
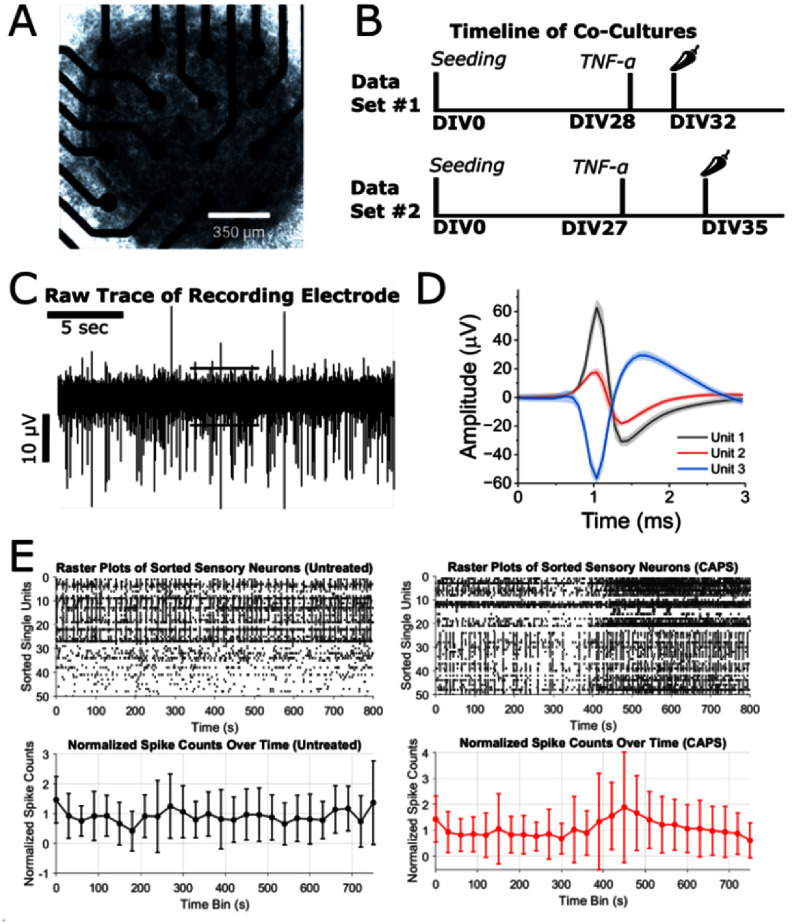
Experimental setup and single unit neuronal data (a) phase contrast image of hiPSC SN co-cultures on an MEA. (b) Timeline of cell cultures. Chili pepper represents capsaicin addition. (c) Raw trace of MEA recording with adaptive threshold detection. (d) Averaged waveforms of sorted units. (e) (Top) Raster plots that represent fifty sorted single unit spiking and (Bottom) thirty-second binned spike counts normalized by dividing all counts by the median of the first two minutes of the recording. (Left) Untreated co-cultures and (Right) CAPS-treated co-cultures. CAPS administered at t = 300 s into the recording.

Single unit sorting has been established as a means for discriminating the number of EAP signal generators (i.e. individual neurons) for more than 30 years [12]. Its use is ubiquitous and necessary when using brain-machine interfaces (BMIs) that rely on intracortical electrode array recordings [13]. Simply put, an EAP waveform shape is dependent on the cell’s distance from the working electrode, its polarity relative to the working/reference electrode, the organelle from which the recorded signal originates, and its expression of various voltage-gated ion channels [14]. These factors coalesce into an identifiable EAP waveform shape for each neuron and potentially each phenotypic state, meaning that signals from several individual neurons can be resolved from a single electrode’s recording. While single-unit sorting has proven invaluable in neural decoding for BMIs, we believe this approach to be important for enhanced extracellular electrophysiology-based phenotypic drug screening, especially in heterogeneous neuronal cultures/co-cultures as sorting allows for observable single-cell responses in a relatively complex but controlled system. Importantly, there are other means for identifying numbers and types of SNs in a co-culture (e.g. immunocytochemistry (ICC) or single cell RNA-seq), but these methods are either potentially damaging or terminally destructive and, in either case, do not allow continued monitoring or post hoc correlations of phenotypic activity. This makes it difficult to establish a ground truth, so unsupervised learning is necessary to discover patterns in the neuronal recordings following exposure to subtype-specific compounds—which is possible to administer with our MEA platform (figure 1(a)). Unsupervised methods for phenotype discovery have been applied to a broad range of applications related to phenotyping other cells [15–19] and will be the method for generating the SN subtype labels using MEA-recorded electrophysiological response evoked with known agonists or antagonists.

Here, we have co-cultured hiPSC SNs and glia on MEAs and evoked an inflammation-like state through the addition of inflammatory cytokine tumor necrosis factor alpha (TNF-*α*). We set aside two random wells of cultured neurons for a test set and used the remaining six wells as training/validation data and labeled nociceptive single units (i.e. individual neurons) using an unsupervised univariate statistical approach based on capsaicin (CAPS) evoked absolute differences in mean firing rate (MFR). We then compared the performance of five classifiers for their ability to predict whether neurons were nociceptive using the baseline activity of the pharmacologically-labeled cells. The classifiers being compared consisted of a feed forward neural network (FNN), logistic regression classifier (LOG), linear discriminant analysis (LDA) Bayesian classifier, decision tree (Tree) and k-nearest neighbor classifier (KNN). Through 10-fold cross-validation, it was found that no single model outperformed the others. Nonetheless, a trained RUS-boosted ensemble of decision trees was then evaluated against the baseline neuronal activity from left-out wells and presumptive nociceptors and non-nociceptors were classified at an AUC-ROC of 0.8677.

## 2. Materials and methods

### 2.1. SN co-cultures and MEA recordings

HiPSC derived SN and glia co-cultures were seeded on pre-treated 48-well MEA plates (Axion Biosystems, Atlanta, GA, Software Version 3.10) as previously described [4]. In each well, there are 4-by-4 electrodes with a diameter of 50 µm and a pitch of 350 µm. Briefly, Polyethylenimine and laminin (Thermo Fisher, Waltham, MA)-treated wells were seeded with a combination of 30 000 iPSC SNs (Anatomic, Inc., Minneapolis, MN) and 20 000 iPSC astrocytes (BrainXell, Inc., Madison, WI) in a 5 µl volume droplet, manually placed at the center of the well. Cultures received 200 µl of media consisting of Anatomic’s Sensor-MM without Inhibitors plus 1:1000 BrainXell astrocyte supplement. On day in vitro 27, 100 µl of media was removed and 100 ng ml^−1^ (TNF-α, Sigma-Aldrich, #SRP3177) diluted in 100 µl of media was added to the cells to yield a concentration of 50 ng ml^−1^ in each well to evoke an inflammatory state. Electrical potential measurements were carried out across all 768 electrodes simultaneously at a sampling rate of 12.5 kHz. Data were filtered using an analog 1-pole Butterworth band-pass (250–3000 Hz, 1000x Gain) and EAPs were identified by continuous filtered data crossing an adaptive threshold (± 5.5σ). For each EAP, 3.04 ms time windows centered about the threshold crossing were extracted from the recording and stored as a waveform (or EAP shape) with its corresponding time stamp. EAPs were manually sorted into single units based on identifiable clusters in 2D principal component space using Plexon’s Offline Sorter (Dallas, TX, Version 4.4.0).

### 2.2. Response and baseline feature extraction

After a five-minute recording period for all wells, 0.2 µl droplets of 982 µM CAPS transient receptor potential vanilloid-1 (TRPV1 agonist, Sigma–Aldrich, #211275) diluted in 95% ethanol (Decon Labs, #2801) was added to all fourteen wells (n = 622 cells) to yield a concentration of 1 µM. CAPS-treated cells were evaluated for labeling by identifying thirty-second periods of highest neuronal activity, identified by parsing the baseline and treatment periods into thirty-second bins, and for each cell, selecting the bins that exhibited the greatest number of spikes. The absolute difference in MFR was calculated by subtracting the MFR of the baseline bin from the MFR of the treatment bin. We opted to observe absolute differences in MFR instead of using percentage change—a good standard practice in studies that label individual samples undergoing treatment [20]. However, this is a study limitation that will be addressed in the discussion.

While the thirty-seconds representative bins were used to assign absolute MFR changes, we utilized the entire baseline recording period to quantify the functional phenotype used to train the classifiers. This was necessary for minimizing the likelihood that the training data influences the ground truth definition. Four features were chosen to represent the phenotype of individual neurons: MFR, interspike-interval coefficient of variation (ISICV), median pair-wise synchrony, and the skewness of pair-wise synchrony. Pair-wise synchrony was estimated using PySpike, a validated approach for estimating the correlation of spike trains between neurons [21]. This approach allows for the estimation of spike correlation between two neurons within a well. The median and skewness of all pair-wise comparisons informs the relationship of a given neuron to the larger network. Prior to deploying the machine learning models, corresponding features were statistically compared to determine whether there were observable differences between the presumptive subtypes (Mann–Whitney U test, α = 0.05) using MATLAB (MathWorks, Natick, MA, Version 24.1.0).

### 2.3. Unsupervised classifier for approximating functional subtype labels

By referencing untreated SN co-cultures, we employed a kernel density estimation (KDE) model that classifies cells based on compound-mediated response. Using MATLAB, we employed KDE with untreated neuronal data (n = 621) to estimate the distribution of random changes in MFR between maximum bins collected before and after pseudo-treatment. KDE calculates the probability density for each CAPS-treated neuron by assigning weights based on its distance from all other points in the untreated pseudo-treatment data. Each weight is adjusted using a smoothing parameter, bandwidth, which was calculated using Silverman’s rule of thumb [22]. The sum of the weights produced the final density estimate for each treated neuron. This KDE procedure is then repeated for the untreated dataset, where each neuron is assigned a density estimate based on its distances from other neurons in the same pseudo-treatment dataset. Using a percentile-based approach, we classified neurons as ‘nociceptors’ if their KDE values in the CAPS-treated response data fell below the 10th percentile of the KDE distribution for the untreated response data. Below is the equation that represents KDE (equation (1)).

\begin{equation*}{\hat f_h}\left( x \right) = \frac{1}{{nh}}\sum\limits_{i = 1}^n K\left( {\frac{{x - {x_i}}}{h}} \right).\end{equation*}

Equation (1). Kernel density estimation, where *n* is the number of untreated samples, *h* is the bandwidth, *x* is a vector that contains a CAPS treated cell’s absolute difference in MFR, *y_i_* is the *i*_th_ vector that contains the absolute difference of the *i*_th_ sample in the untreated co-culture data. *K* is a Gaussian kernel function that returns a weight inversely proportional to the distance between a *x* and *y_i_*.

### 2.4. Training and evaluating subtype classifier using supervised learning

Cells were labeled based on their response to nociceptor agonist CAPS using KDE to identify neurons that were likely to be nociceptive. The baseline data from these cells were compiled into a single array of training data composed of the previously mentioned feature set (n = 622 cells, 36.82% nociceptors). We compared the AUC-ROC of five models through 10-fold cross validation [23]. These models were feed FNN ensemble, LDA, LOG, RUS-boosted decision tree ensemble [24] (Tree), and KNN feature subspace ensemble [25]. Aside from the Tree ensemble (which under samples the majority class), the minority class was weighted higher than the majority class during model training. All models were MATLAB-provided classifiers, parameterized using Bayesian optimization [26, 27] testing standard hyperparameters associated with each unique classifier. Tables indicating the hyperparameters tested are included in supplemental materials (supplementary table 1). The AUC-ROC was calculated using MATLAB’s ‘perfcurve’ function, which provides a measure of the probability that a given model ranks the positive class higher than the negative class. To determine the optimal model, we performed group-wise comparisons between the classifiers’ AUC-ROC based on a Kruskal–Wallis test (α = 0.05) using MATLAB. The model that exhibited the best empirical performance was tested again using a test set that consisted of one well from each dataset that was excluded from the validation/training process. If it was found that none of the classifiers statistically outperformed the other, we opted to demonstrate the performance of the tree due to its ability to fit a non-linear decision boundary while also providing interpretability as feature importance metrics are readily available [28]. In addition to accuracy (true predictions divided by the total number test samples), we calculated the F1 scores for both classes (described here [29]), and the AUC-ROC. Furthermore, we performed principal component analysis (PCA) on the labeled training and test data to qualitatively observe the two subtypes in two dimensions.

## 3. Results

SN subtypes were labeled by recording EAPs from SN/glia co-cultures and determining the pharmacological response to a nociceptor agonist. To pharmacologically identify presumptive nociceptors among our heterogeneous SN/glia co-culture (figure 1(a)), we cultured 30/20k hiPSC SNs and glia on multi-well MEA plates and exposed them to 1 µM CAPS, a known agonist of nociceptor activity (figures 1(b)–(d)) show our ability to record EAPs from single electrodes with high signal to noise ratios and then sort single units (i.e. single neurons’ activity) based on characteristic waveform shapes, respectively. Figure 1(e) shows our ability to qualitatively observe CAPS-evoked changes in activity.

**Figure 2.**
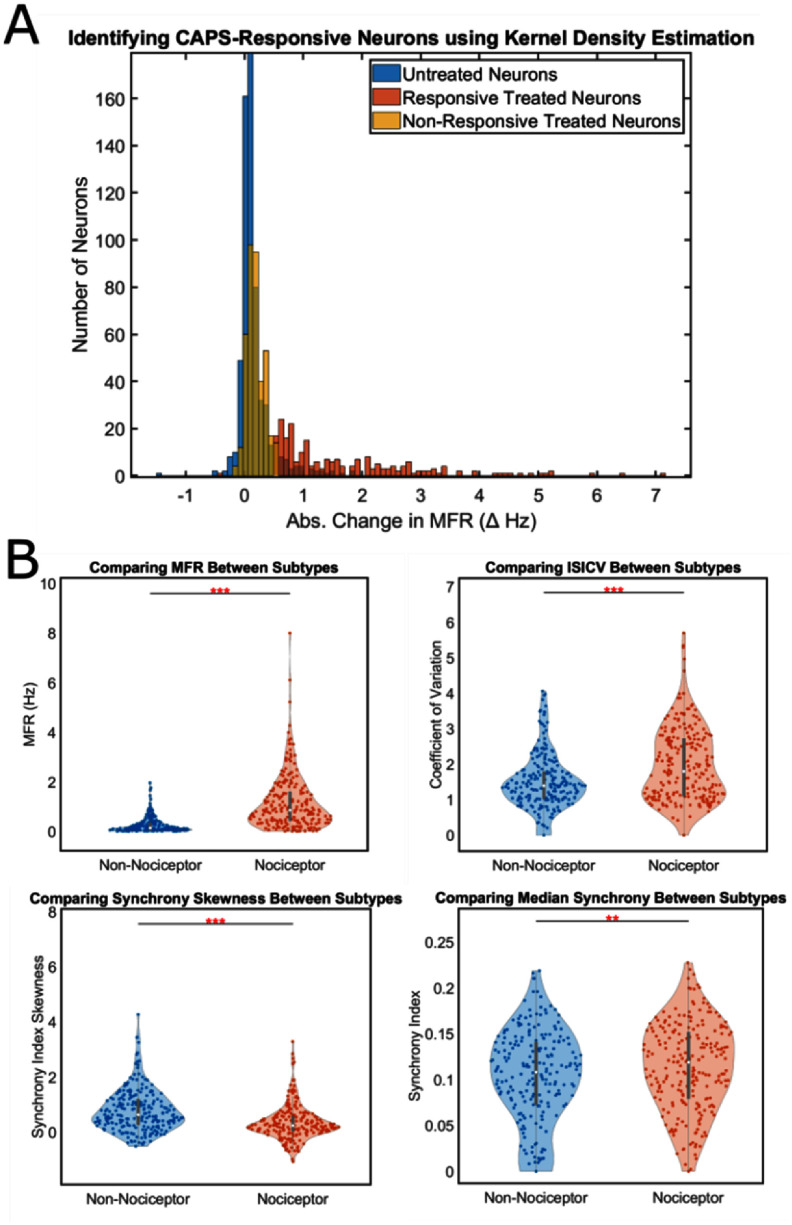
Establishing ground truth for SN subtypes. (a) Evoked-response data of cells exposed to CAPS compared to the natural transience of untreated SNs. (b) Simple statistical comparisons between baseline features of CAPS-labeled subtypes (Mann–Whitney U test, α = 0.05). ** and *** signify p-values less than 0.01 and 0.001, respectively.

Compared to untreated SNs, 36.82% of CAPS-treated cells exhibited higher MFR (figure 2(a), red, n = 229). The KDE between the baseline variability and the compound-treated response differences was calculated for each SN in the compound-treated group. The probability density estimates that fell outside the 90th percentile were labeled as presumptive nociceptors. Otherwise, cells that did not were considered non-nociceptors (figure 2(a), yellow, n = 393). The x-axis represents the differences in MFR, the y-axis number of neurons in a given range of MFR differences, and the color represents the treated responsive neurons (red), treated non-responsive neurons (yellow), and untreated neurons (blue). In general, CAPS-treated neurons had a positive response—and we used those responses to label cells that express TRPV1. 36.82% of SNs exposed to CAP were labeled as TRPV1 positive (nociceptor) (table 1). Prior to testing the machine learning models, we compared each of the features between the two subtypes using the Mann–Whitney U Test (α = 0.05). It was found that the presumptive nociceptors exhibited statistically higher MFR, ISICV, and median pair-wise synchrony. The distribution of pair-wise synchrony indexes for non-nociceptors were generally skewed right (figure 2(b)).

**Table 1.** Summary of HiPSC SN subtype classification.

Nociceptor proportion	Training set nociceptor proportion	Test set nociceptor proportion	Training set sample size	Test set sample size	Test AUC-ROC
36.82%	36.69%	37.88%	556	66	0.8766

**Figure 3.**
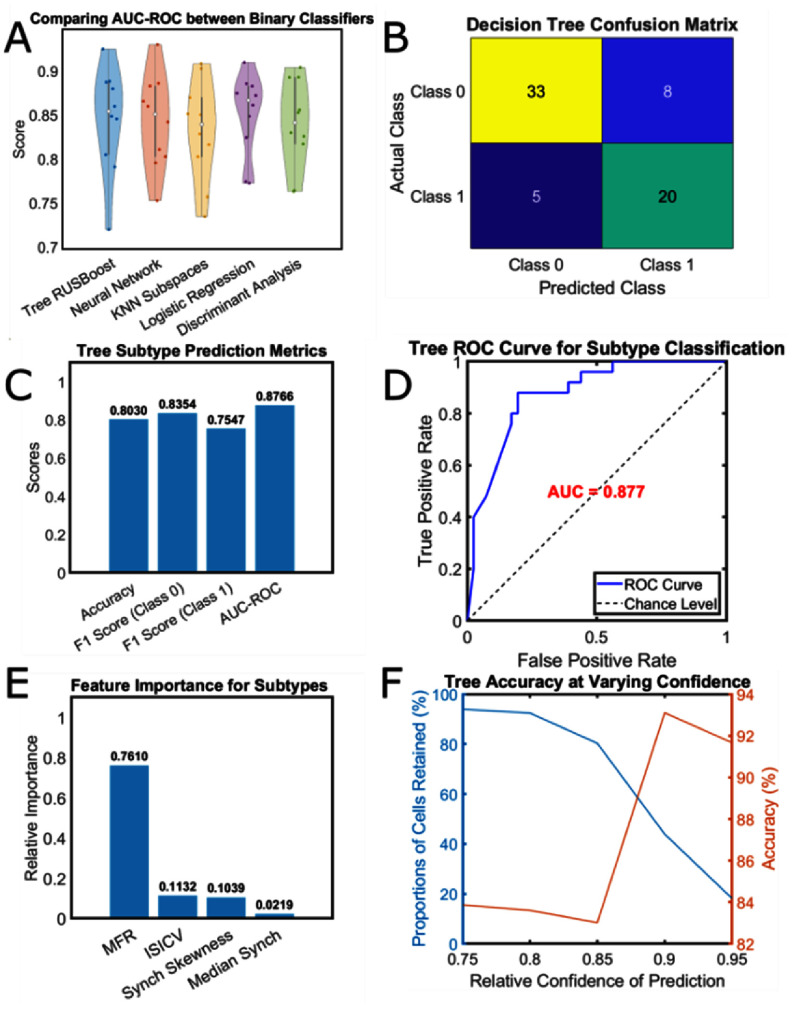
Results of subtype classification (a) group comparisons of AUC-ROC for five classifiers Kruskal–Wallis (α = 0.05) following ten-fold cross-validation. (b) Tree confusion matrix of the left-out test data that was not used during cross-validation. (c) Performance metrics of the Tree for the left-out test set: accuracy, F1 score (Class 0), F1 score (Class 2), and AUC-ROC. (d) Receiver operating characteristic curve of Tree predictions of nociceptors. The decision threshold is varied. (e) Tree feature importance for classifying subtypes. (f) Curves demonstrating the trade-off between cells with high-confidence predictions and the accuracy of those predictions as we increase the threshold for Tree prediction confidence.

All classifiers resulted in statistically similar classification AUC-ROC values based on 10-fold cross-validation (figure 3(a)). Figure 3(b) shows the final evaluation of the Tree against left-out wells balanced for each subtype. For the balanced test set, the accuracy, non-nociceptor F1 score, and nociceptor F1 score was 80, 84, and 75% respectively (figure 3(c)), and the AUC-ROC was 0.88 (figure 3(d)). By observing the feature importance of the trained Tree, it was found that MFR was the most meaningful feature for nociceptor classification (figure 3(e)). The Tree achieved an accuracy of 84% for high confidence predictions (⩾ 80% confidence) at the expense of losing 6.07% of the test set (figure 3(f)).

**Figure 4.**
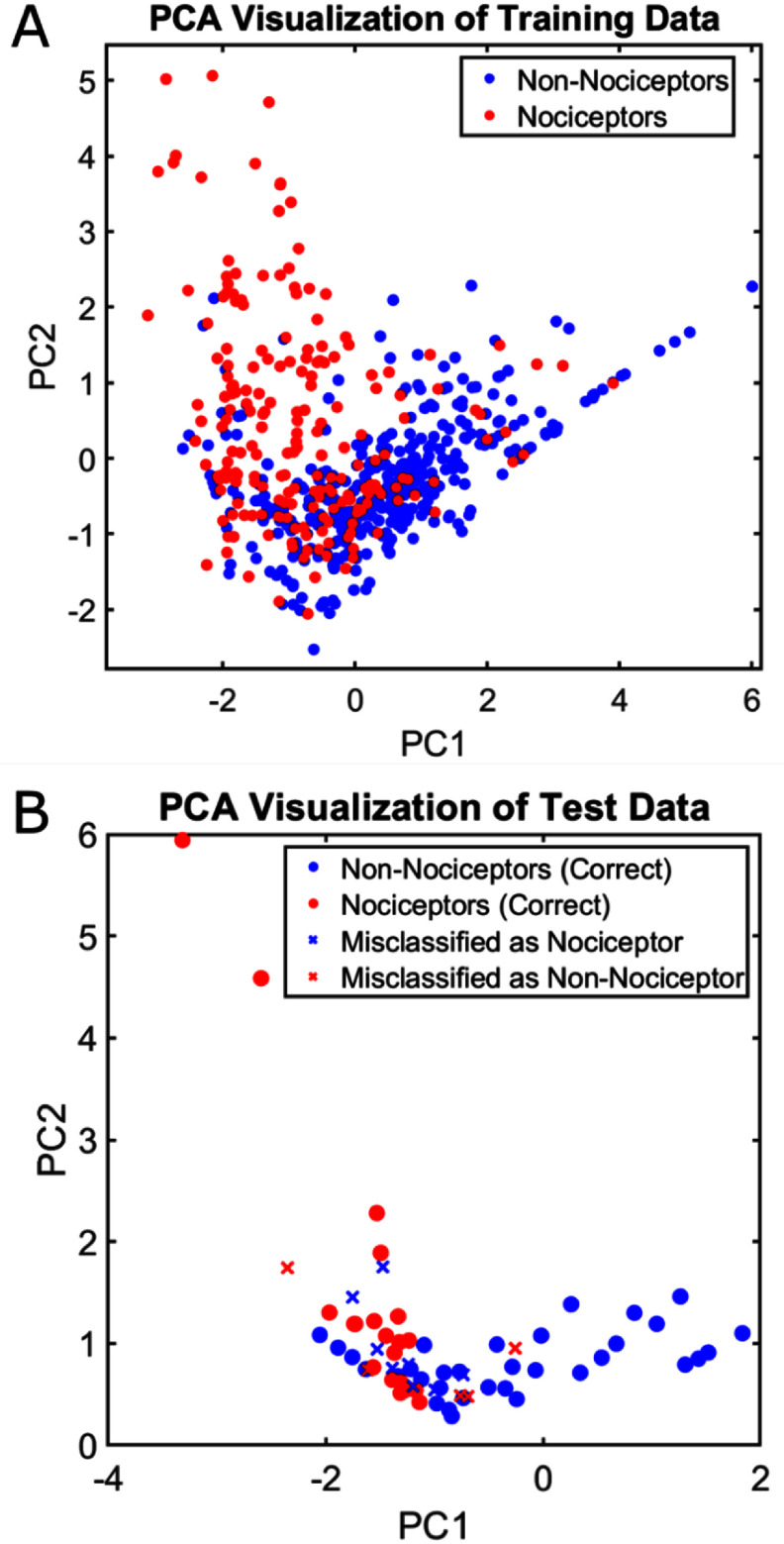
Training/validation and unseen test data represented using principal components (a) first two principal components of the four baseline SN features used to train and validate the machine learning models. The red marker represents a SN that was responsive to CAPS while the blue marker represents SNs that were not responsive to CAPS. (b) The left-out unseen test data, with marker shapes corresponding to whether it was correctly predicted by the Tree classifier, and the color representing the true subtype label. The data in 4B was generated using neurons not included in cross-validation or training. An ‘x’ signifies an incorrectly predicted phenotype, while circles represent a correct prediction.

The test set (wells reserved for optimal model evaluation) exhibited similar relationships between subtype and baseline activity as observed in the training and validation sets (figure 4(a) vs. Figure 4(b)), which are represented below using PCA. The Tree was able to predict the subtypes in the test set with notable accuracy (figures 3(b)–(d)), and the specific predictions at specific baseline features are demonstrated in figure 4(b).

## 4. Discussion

In our previously published work, we demonstrated a novel and highly effective stand-alone assay based on iPSC derived SNs and glia co-cultures and MEA recordings [4]. However, SN heterogeneity, both in our culture and other heterogeneous systems/models, while important to maintain, may obscure pharmacological ‘hits’ due to well-wide averaging across neuron populations. Previous studies have employed ICC and/or transcriptomic techniques to identify neuronal subtypes in the dorsal root ganglion (DRG). This has led to the categorization of at least 19 SN subtypes in DRG [30] and 17 subtypes in rodent DRG [31]. Even amongst nociceptors, there are at least five sub-categories in human DRG [32]. It is well established that direct contact between neurons and glia improves long-term viability and modulates functional responsiveness of cultured neuronal networks [4]. It is reasonable to assume that retention of SN subtypes that differentially secrete growth and guidance cues may also mediate the differentiation and phenotypic activity of other SN subtypes. This underlines the importance of maintaining neuronal heterogeneity in neuronal models of function and disease, generally, and in the case of SN co-cultures, particularly.

Our work demonstrates that machine learning can be employed to predict SN subtypes using baseline spontaneous recording information following culture-dependent pharmacological training data. This ability will enhance novel analgesic screening but also has implications for other phenotypic screening based on MEA activity and heterogeneous cultures, including those carried out for seizures [33], ALS [34], and Alzheimer’s [35], provided pharmacologically labeled training sets were created based on other neuronal subtype activation.

As expected, we were able to record EAPs from hiPSC SN/glia co-cultures with excellent signal-to-noise ratios. For this study, we also performed manual sorting of single units based on separation in 2D principal component space (figure 1(b)). While manual sorting is common practice in the fields of in vitro and in vivo neural recordings [36], it is open to bias for over- or under-counting the number of units on a given electrode. This is less important for our application since 1) we did not have an expectation for unit size/shape/number in relation to cell type and 2) the basis for activity discrimination was based on evoked absolute change and not on raw number of EAPs/units per electrode. Figure 1(C) also illustrates responsive as well as unresponsive units to the applied agonists/antagonists, suggesting a functionally heterogeneous SN population. This has been confirmed in our previous study using ICC [37] and further suggested by transcriptomics studies [38].

While previous works have employed machine learning techniques for discerning neuronal subtypes, this workflow (figure 1(a)) is the first of its kind as functional nociceptive subtype classification following pharmacological labeling has not been demonstrated for SNs—especially iPSC-derived SNs. Our findings show that there is a subpopulation of SNs in our cultures that following CAPS treatment do not exhibit differences in behavior that exceed the normal transience of untreated SNs (figure 2(a)). To establish ground truth classification of presumptive nociceptors, we employed an un-supervised approach using the thresholded response to CAPS. This compound competitively binds to TRPV1 [39], serving as an effective agonist of presumptive nociceptor activity. It should be noted that many peripheral and central neuronal subtypes express TRPV1 channels at different levels [40]. As such, CAPS may evoke a response in non-nociceptors at sufficiently high concentrations [41]. However, CAPS has been used repeatedly in vivo [42] and in vitro [43] as a positive control compound for nociceptor identification/targeting. While more targeted classification methods, such as single-cell RNAseq, can be deployed to identify the existence and abundance of SN cell types, no non-destructive methods are available for cell type classification that pre/conserve phenotypic activity. Treated cells with differences in MFR that yielded KDEs outside of a 90th percentile relative to untreated cells were considered presumptive nociceptors (figure 2(a)). We landed on this metric because change in spike-rate is a functional characteristic that has been previously described to be strongly associated with pain signaling [44]. Most notably, the baseline activity of this non-nociceptive subpopulation differs from the baseline activity of presumptive nociceptive-population as these cells had significantly lower MFR, lower inter-spike interval coefficient of variations, lower median pair-wise synchrony, and higher pair-wise synchrony skewness (which implies lower pair-wise synchronous relationships) (figure 2(b)).

Absolute differences, rather than a relative- or percentage-change in MFR, were used to label the cells. The most appropriate way to calculate treatment effect in biomedical studies is disputed and application dependent [45]. We elected to compare absolute versus relative changes (i.e. fold change) as it has been reported that absolute change approaches have higher statistical power in correlated data (50.7% vs. 45.1% [20]) versus those based on relative change. Furthermore, Chen *et al* [46], a study highly related to our own, functionally characterized neuronal subtypes in the auditory cortex using absolute differences in firing rate. Ground truth classification resulted in 37% of CAP-treated single units (i.e. single neurons) being labeled as presumptive nociceptors. While commercial iPSC SNs in general, and specifically those used in this study, do not claim to represent all varieties of SNs found in the human DRG, this percentage is reasonably consistent with peptidergic nociceptor population percentages observed in both rodent (35% TRPV1+ [47]) and human (54% TRPV1+ [47]) DRG studies based on ICC or spatial/single-cell transcriptomics. The discrepancy between our approach and the true human TRPV1+ population is most likely the result of considering near-quiescent SNs with marginal CAPS-evoked increases in activity as non-nociceptive (i.e. minor absolute change but large relative change). Moreover, our study relies on a functional response of ion channels known to be differentially expressed across cells even within a given cell type. In short, this may be a matter of thresholding differences between the studies. This supposed second form of nociceptor makes up about 10% of cells considered non-nociceptors in this study, making this a limitation to our labeling technique. Nonetheless, we have included the results of training classifiers to discriminate these types of cells in the supplemental (supplementary figure 1) section and will address the proposition that there is more than one type of nociceptor in future studies.

It was found that none of the models significantly outperformed each other (figure 3(a)). Classifiers were compared using 10-fold cross validation—breaking the training data into ten random folds and evaluating the AUC-ROC on each fold after training on the remaining nine. This produced ten AUC-ROC values for each of the models, and this was group-statistically compared using a Kruskal–Wallis test. Nonetheless, the ensemble of decision trees (Tree) is a priori, a well-suited approach to accommodate both non-linearities and class imbalance in the data. The ensemble of decision trees can fit a complex decision boundary for discriminating against the two classes while also being ‘boosted’ to under sample the majority class for each learner in its ensemble. Furthermore, it can provide descriptive information about the relative importance of a feature within the training data. In contrast, LDA and LOG are less equipped for fitting complex decision boundaries, which was not necessarily a limitation here, but may become relevant as we incorporate more than two functional subtypes into the classification problem. The default KNN cannot report feature importance, which is a crucial piece of information in these types of exploratory machine learning studies. FNNs, while being able to fit a complex decision boundary, also cannot provide feature importance by default and the model itself is fairly opaque when attempting to understand reasoning behind a prediction. Despite our relatively small dataset, the Tree generalized well to the left-out wells reserved for testing, and this was reflected in the accuracy and AUC-ROC (80% and 0.88 respectively, figures 3(c) and (d)). While there is room for improvement, the accuracy of the Tree falls near the performance range of classifiers that perform similar tasks, such as Bardy *et al* iPSC neuronal functional state classifier—which incorporates transcriptional and electrophysiological data (83%–92% accuracy [48]). Furthermore, the Tree provided higher accuracy high-confidence predictions (83.87% accuracy, c ⩾ 80%, figure 3(f)), while retaining a considerable portion of the dataset (93.93% cells discovered at c ⩾ 80%). This is an important consideration in general, but specifically in the case of our phenotypic assays since scalable hiPSC generation and differentiation is expensive in both time and cost. Moreover, single unit yields within wells may range from as low as 11–33 depending on primary versus hiPSC cultures, meaning that cell classification exclusions are potentially prohibitive depending on electrode and unit numbers. For example, our published assay requires a minimum of 4 units per well and 4 wells per treatment to remain statistically viable as a stand-alone assay. Trading accuracy/confidence for sample numbers will reach a point of diminishing returns.

Following the final evaluation of the Tree, it was revealed that the MFR was the most important feature regarding binary classification. This is broadly consistent with previous literature attempting to map the relationship between phenotypic states of neurons to their pattern of activity [49]. In future studies, not only do we want to explore the prospect that there are more than two functional subtypes in the hiPSC co-cultures, but explore more complex architectures equipped to handle correlation between training samples. Here, we used the amount of correlation (synchrony) as one of the features—but there are more sophisticated techniques for handling this, such as using a graph neural network.

## 5. Conclusion

Phenotypic screening leverages cell types and measurements relevant to disease phenotypes to initially screen libraries of compounds for efficacy. Subsequent target deconvolution can then be used to determine mechanistic pathways and safety profiles. By integrating a classifier to predict subtypes upstream to phenotypic analysis, the process of target deconvolution could be greatly improved as we can apply ‘weights’ to neurons based on their presumptive identity and allows selective analysis of their pharmacological response. Here, we have demonstrated the feasibility of classifying presumptive nociceptors from a general population of iPSC SNs based only on their spontaneous baseline activity. This suggests that we can, in a non-destructive way, down-select nociceptor or non-nociceptor signals in our analysis to identify cell-type specific drug effects. Going forward, more recordings of SN data coupled with an optimized feature set will be necessary to increase the significance of this machine learning application. Despite the limited sample size, the Tree performed well with unseen data (0.8766 AUC-ROC) and is a feasible approach for enriching our phenotypic screening platform.

## Data Availability

The data cannot be made publicly available upon publication because they are not available in a format that is sufficiently accessible or reusable by other researchers. The data that support the findings of this study are available upon reasonable request from the authors.
